# Oral hyaluronan relieves knee pain: a review

**DOI:** 10.1186/s12937-016-0128-2

**Published:** 2016-01-27

**Authors:** Mariko Oe, Toshiyuki Tashiro, Hideto Yoshida, Hiroshi Nishiyama, Yasunobu Masuda, Koh Maruyama, Takashi Koikeda, Reiko Maruya, Naoshi Fukui

**Affiliations:** 1 R&D Division, Kewpie Corporation, 2-5-7, Sengawa-cho, Chofu-shi, Tokyo Japan; 2 Tokyo Yamate Medical Center, 3-22-1 Hyakunin-cho, Shinjyuku-ku, Tokyo Japan; 3 Gate Town Hospital, 1-6-19, Sekimachi-kita, Nerima-ku, Tokyo Japan; 4 Shiba Palace Clinic, 1-9-10 Hamamatsucho, Minato-ku, Tokyo Japan; 5 SOUKEN Corporation, 1-9-10, Hamamatsu-cho, Minato-ku, Tokyo Japan; 6 Department of Life Science, Graduate School of Arts and Sciences, The University of Tokyo, 3-8-1 Komaba, Meguro-ku, Tokyo Japan

**Keywords:** Hyaluronan, Hyaluronic acid, Dietary supplement, Knee, Joint, Osteoarthritis

## Abstract

Hyaluronan (HA) is a component that is particularly abundant in the synovial fluid. Randomized, double-blinded, placebo-controlled trials carried out between 2008 and 2015 have proven the effectiveness of HA for the treatment of symptoms associated with synovitis, and particularly, knee pain, relief of synovial effusion or inflammation, and improvement of muscular knee strength. The mechanism by which HA exerts its effects in the living body, specifically receptor binding in the intestinal epithelia, has gradually been clarified. This review examines the effects of HA upon knee pain as assessed in clinical trials, as well as the mechanism of these effects and the safety of HA.

## Introduction

The number of patients with osteoarthritis (OA) is increasing in developed countries. In the US, 43 million patients were estimated to have OA in 1997, and this figure is projected to grow to more than 60 million by 2020 [1].

Severe OA is treated by osteotomy or artificial joint replacement, whereas conservative treatments include intra-articular injection of hyaluronan (HA) [2]. The injections improve symptoms, although they represent a mental burden and the risk of infection for patients who need to visit the hospital regularly to receive these painful injections.

Meanwhile, dietary supplements such us HA, glucosamine, and chondroitin are sold as health foods, largely targeting problems with the knee. The effects of low-dose HA treatment for knee pain has been the source of much research (for example, a dose of HA not more than 240 mg/day; Table 1) compared with glucosamine (1500 mg/day) and chondroitin (675 mg/day) [3]. HA dietary supplements impose a small burden on patients.

**Table 1:** Summary of the knee pain-improving effects of ingested hyaluronan

Study designs	Materials and Methods	Subjects	Results	References
Randomized, double-blind, placebo-controlled trial	HA mixture at 630 mg (HA 60 mg; MW <5 k) daily for 2 weeks	24 patients with knee pain (in Japan)	Significant improve of knee pain and discomfort	[34]
Randomized, double-blind, placebo-controlled trial	HA mixture at 80 mg (HA 48 mg; MW 1000 k) daily for 2 months	20 patients aged ≥40 years with knee OA (in USA)	Significant improve from baseline for bodily pain bodily pain subscale and physical component summary.	[35]
Randomized, double-blind, placebo-controlled trial	HA at 240 mg (MW 900 k) daily for 8 weeks	26 patients aged 50 ~ 65 years with knee pain (in Japan)	Significant improve of knee pain and stiffness	[36]
Randomized, double-blind, placebo-controlled trial	HA at 200 mg (MW 900 k) daily for 8 weeks	25 patients with knee OA [WOMAC pain score > 10] (in USA)	Significant improve of tatal WOMAC score and activity of daily living	[37]
Retrospective cohort study, PCT-controlled trial	HA mixture at 80 mg (HA 48 mg; MW N/A) daily for 6 months	69 patients with knee OA and synovitis (in Spain)	Significant improve of synovial effusion and knee pain	[38]
Randomized, double-blind, placebo-controlled trial	HA mixture at 630 mg (HA 60 mg; MW <5 k) daily for 4 months	40 patients with knee OA and synovitis (in Japan)	Significant improve of pain/step-up and -down function and aggregate total symptoms	[39]
Randomized, double-blind, placebo-controlled trial	HA mixture at 2520 mg (HA 72 mg; MW <5 k) daily for 12 weeks	29 patients with knee OA and synovitis (in Japan)	Significant improve of bone metabolism marker	[40]
Randomized, double-blind, placebo-controlled trial	HA at 200 mg (MW 900 k) daily for 12 months	38 patients with knee OA (in Japan)	Significant improve of Health condition	[41]
		21patients aged ≦70 years with knee OA (in Japan)	Significant improve of total JKOM score, pain and stiffness in the knee and general activities	
Randomized, double-blind, placebo-controlled trial	HA mixture at 80 mg (HA 52 mg; MW N/A) daily for 90 days	40 healthy individuals with mild joint discomfort (in Spain)	Significant improve of joint mechanics and muscle function	[42]
Meta-analysis included in two randomized, controlled, double-blind, placebo-controlled trials	HA mixture at 80 mg (HA 48 mg; MW N/A) daily for 3 months	148 healthy individuals with mild knee pain (in Spain)	Significant improve of muscle function, synovial effusion and reduces pain	[43]
Randomized, double-blind, placebo-controlled trial	HA mixture at 80 mg (HA 52 mg; MW N/A) daily for 90 days	68 healthy individuals with mild joint discomfort (in Spain)	Significant improve of articular pain, synovial effusion and knee muscular strength	[44]
Randomized, double-blind, placebo-controlled trial	HA mixture at 80 mg (HA 56 mg; MW N/A) daily for 3 months	40 patients with knee OA (in USA)	Significant improve of total WOMAC score and knee pain	[45]
Randomized, double-blind, placebo-controlled trial	HA mixture daily for 4 weeks (HA 225 mg daily for first 2 weeks, HA 150 mg daily for last 2 weeks; MW 2500 k ~ 2800 k)	72 patients with knee pain (in USA)	Significant improve of knee pain	[46]

The average wholesale price for five vials of intra-articular HA injection (Hyalgen®, Fidia Farmaceutici S.p.A., Italy) is 661 USD, which is effective for 6 months [4]. This equals to 110 USD per month. In contrast, the cost of effective HA dietary supplements for one month is 50 USD (Play Again Now®, Viscos LLC., USA). There have been no reports regarding the cost-effectiveness of intra-articular injection versus dietary supplements. However, there are some advantages for consumers with regard to HA dietary supplements, when one considers the potential risks and benefits.

In 2004, Bucci and Turpin reported a review article on the effectiveness of dietary supplements containing HA in the US; however, the report does not mention randomized, double-blind, placebo-controlled trials [5]. Therefore, this review discusses the efficacy of ingested HA in treating knee pain based on data from randomized, double-blind, placebo-controlled trials as well as the mechanism of action and safety of dietary HA.

## Characteristics of HA

**Fig. 1:**
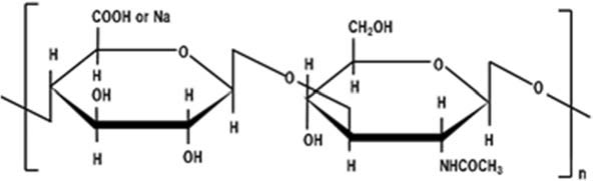
The structure of hyaluronan

HA is a high molecular-weight polysaccharide composed of repeating polymeric disaccharides of d-glucuronic acid and *N*-acetyl-d-glucosamine (Fig. 1) [6]. All vertebrates and few microorganisms synthesize HA in vivo. In human beings, HA is present in every connective tissue and organ such as skin, synovial fluid, blood vessels, serum, brain, cartilage, heart valves, and the umbilical cord [7]. In particular, synovial fluid has the highest concentration of HA anywhere in the body at 3–4 mg/mL [8].

HA is a polysaccharide with a mean molecular weight (MW) ranging from several hundred to several millions, and thus, it has high viscosity in water. The viscosity of synovial fluid is attributable to HA and serves as a lubricant for joint movements, resulting in a coefficient of friction of nearly zero in joint cartilage [9]. It is known that patients with OA have diminished HA concentrations in their synovial fluid [10]. To restore the decreased levels of HA and treat OA, HA intra-articular injections are widely used. The functions of HA include preventing cartilage denaturation [11, 12], protecting the outer layer of cartilage [13–20], blocking synovial inflammation [21, 22], increasing chondrocyte density [23], promoting synovium metabolism [24], normalizing synovial fluid [25], and treating sharp pain [26]. The mechanism by which HA diminishes pain has been reported. HA intra-articular injections decrease the levels of inflammatory substances such as prostaglandin E2 resulting in reduced pain [27, 28]. Therefore, HA is known to have a strong relationship with knee joint health.

## Mechanisms of oral HA treatment for knee pain

It is generally believed that it is difficult for the body to absorb a polysaccharide. HA is not absorbed into the body as a high-molecular-weight polymer after ingestion. A test using intestinal epithelia model cells (Caco-2 cells) revealed that HA with a MW exceeding 1 × 10^5^ is rarely absorbed. On the contrary, the amount of HA absorbed by Caco-2 cells increases as the MW of HA decreases to 7 × 10^4^, 2 × 10^4^, or 5 × 10^3^ [29]. Kurihara et al. reported that HA is decomposed into 2–6-membered polysaccharides by enteric bacteria, and these polysaccharides are partially absorbed into the body by the small intestine [30]. Following the decomposition of HA by enteric bacteria to a low MW form, free polysaccharides are known to migrate into the joints and other tissues. *Lactobacillus* and *Bzfidobacterirn* have been reported as examples of enteric bacteria that play a critical role in HA absorption [31]. Balogh et al. reported that ^99 m^technetium-labeled HA (MW, 1 × 10^6^) is accumulated by tissues such as joints after oral administration in rats and dogs [32]. The pattern of tissue uptake of radioactivity for ^99m^technetium-labeled HA did not resemble that for ^99m^technetium-labeled pertechnetate (PCT), which was used as a control; this implies that ^99m^technetium-labeled HA did not release ^99m^technetium in tissues.

These studies clearly suggest that HA is absorbed by the body; however further research is needed to clarify the effects of the route of absorption on knee pain relief.

**Fig. 2:**
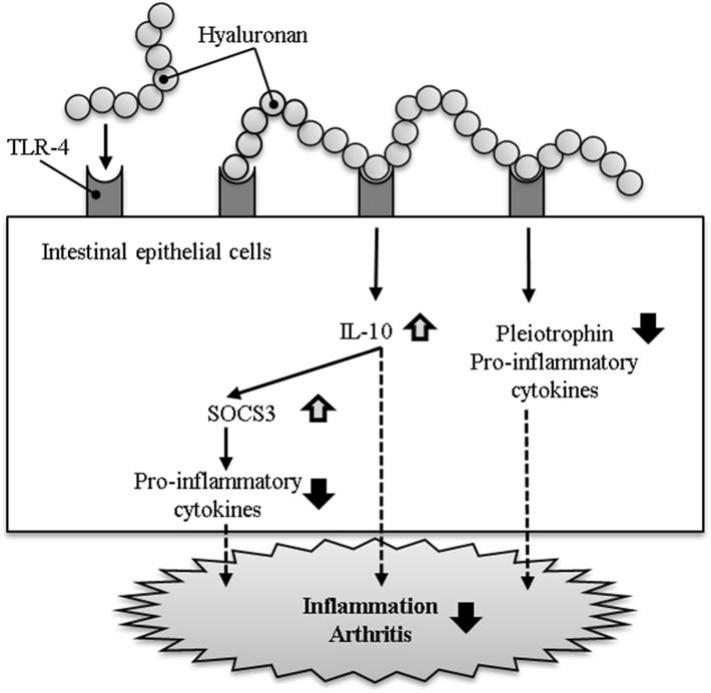
Mechanism of improve the arthritis. Oral administration of hyaluronan modulates inflammation by upregulating suppressor of cytokine signaling-3 expression and down-regulating pleiotrophin expression via Toll-like receptor-4 in intestinal epithelial cells

Another mechanism was clarified by Asari et al. in 2010 [33]. This report identified a signaling cascade in which receptors on intestinal epithelial cells are activated by oral HA which results in decreased pain. HA (MW, 9 × 105; Hyabest® (J), Kewpie Corporation, Tokyo, Japan) was administered orally to MRL-lpr/lpr mice, a Th-1-type autoimmune disease model. Oral HA binds to an intestinal receptor (Toll-like receptor-4;TLR-4). Cytokine array analysis showed that HA enhanced the production of interleukin-10 (IL-10), an anti-inflammatory cytokine. DNA array analysis of tissue from the large intestine showed that HA up-regulates suppressor of cytokine signaling 3 (SOCS3) expression and down-regulates pleiotrophin expression. These results suggest that the binding of HA to TLR-4 promotes IL-10 and SOCS3 expression and suppresses pleiotrophin expression leading to anti-inflammation of arthritis (Fig. 2).

## Clinical trials of oral HA for the treatment of knee pain

We searched the databases up to October 29, 2015; PubMed, the Cochrane Library, Scopus, UMIN-CTR, JDreamIII (in Japanese), and Ichushi Web (in Japanese). The search words used for all databases contained the terms; hyaluronan, intake and knee pain. We selected following 13 relevant reports after full text review [31–43].

Many randomized, double-blind, placebo-controlled trials have demonstrated the effectiveness of dietary HA in alleviating knee pain since 2008 in the US, EU, and Asia (Table 1).

In 2008, a dietary supplement containing HA as the principal ingredient was reported following two clinical trials. As the supplement contains multiple components, the potential effects of ingredients other than HA on knee pain cannot be denied but the effects of the supplement on knee pain were confirmed.

Hatayama et al. [34] treated 24 Japanese patients with chronic knee pain (HA group, *n* = 13; placebo group, *n* = 11, mean age, 47.5) with an HA mixture at a dose of 1800 mg/day (HA content, 60 mg/day) or placebo for 2 weeks. The HA group displayed a significant improvement in knee pain and discomfort compared with the placebo group (*p* < 0.05).

In the US, Kalman et al. [35] treated 20 subjects aged ≥40 years with knee OA (HA group, *n* = 11; placebo group, *n* = 9, mean age, 56.3) with an HA mixture at a dose of 80 mg/day (HA content, 48 mg/day) or placebo for 2 months and their outcomes were evaluated using the index for knee joint pain; the Western Ontario and McMaster Universities Osteoarthritis Index (WOMAC), and the Short Form-36 Acute US Version 2 (SF-36v2) as a quality of life (QOL) index. [REMARK 4] The WOMAC score in both groups revealed significant improvements in knee pain compared with baseline (*p* < 0.05) but the physical function and total symptom scores of the HA group displayed a more drastic improvement than those of the placebo group. On the SF-36 v2, the scores of the HA group were significantly improved versus baseline (*p* < 0.05) for bodily pain and the physical component summary, and the scores were more significantly improved in the HA group than in the placebo group.

In 2009, two clinical trials using highly pure HA (more than 98 % pure) were reported. These trials revealed that the effects of HA-containing supplements on knee pain were attributable to HA.

Iwaso et al. [36] reported that 33 Japanese patients with knee pain (HA group, *n* = 16; placebo group, *n* = 17, mean age, 58.3) were administered HA at 240 mg/day or placebo for 8 weeks and outcomes were evaluated using the Japanese Knee Osteoarthritis Measure (JKOM). The JKOM score was significantly improved compared with baseline in both groups (*p* < 0.01), particularly among 26 patients aged 50–65 years (HA group, *n* = 13; placebo group, *n* = 13). Significant improvements in knee pain and stiffness were recorded in the HA group compared with the placebo group (*p* < 0.05).

Sato et al. [37] reported a study in which 37 Americans with knee OA (HA group, *n* = 20; placebo group, *n* = 17, mean age, 70.8) were orally administered 200 mg/day HA or placebo for 8 weeks with the findings illustrating that the WOMAC score was significantly improved in both groups versus baseline (*p* < 0.05), particularly among the 25 patients with WOMAC pain scores of more than 10 (HA group, *n* = 13; placebo group, *n* = 12). In addition, the WOMAC total score and activity of daily living score were significantly improved in the HA group compared with the placebo group (*p* < 0.05).

In the same year, Möller et al. [38] conducted a retrospective cohort study in Spain in which HA was compared with the analgesic drug paracetamol (PCT). Sixty-nine patients (mean age, N/A) with knee OA and synovitis were administered an HA mixture at 80 mg/day (HA content, 48 mg/day) or PCT for 6 months. Ultrasonography showed that the course of synovitis in the suprapatellar recess was significantly reduced in the HA group compared with the PCT group (*p* < 0.0001), and the number of severe synovial effusion cases were significantly reduced in the HA group compared with the PCT group (*p* < 0.001).

Between 2010 and 2015, eight clinical trials were reported indicating that interest in HA intake was becoming more intense in developed nations.

In 2010, Nagaoka et al. [39] reported that 40 Japanese patients with knee OA (HA group, *n* = 19; placebo group, *n* = 21, mean age, 62.9) were administered an oral HA mixture at 1800 mg/day (HA content, 60 mg/day) or placebo for 4 months and the result illustrated that pain/step-up and step-down function and the aggregate total symptoms of the Japanese Orthopaedic Association clinical trials response criteria were significantly improved in the HA group compared with the placebo group (*p* < 0.05) indicating relief of knee pain.

In 2012, Yoshimura et al. [40] reported that 29 Japanese athletes (HA group, *n* = 14; placebo group, *n* = 15, mean age, 20.0) were administered an HA mixture at 4800 mg/day (HA content, 72 mg/day) or placebo for 12 weeks. The urine levels of the bone metabolic markers N-terminal telopeptides of bone-specific type I collagen were significantly lower in the HA group (*p* < 0.05). This result indicates that oral HA influences knee joint health.

**Fig. 3:**
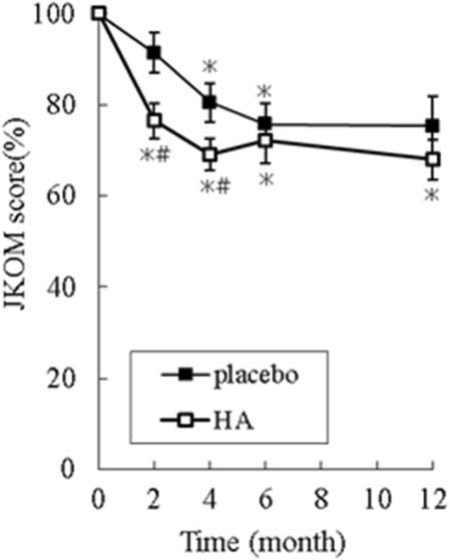
Oral hyaluronan improve knee osteoarthritis: a randomized, double-blind, placebo-controlled trial. Twenty-one subjects (≤70 years of age) were randomly divided into two groups (hyaluronan group, *n* = 11; placebo group, *n* = 10). Variations in the total Japanese Knee Osteoarthritis Measure score relative to baseline are shown. □ hyaluronan; ■ placebo. Values are presented as the mean ± SE. ∗*p* < 0.05 vs. baseline; #*p* < 0.05 vs. placebo group

Tashiro et al. [41] reported a study in which 38 Japanese patients with knee OA (HA group, *n* = 18; placebo group, *n* = 20, mean age, 69.9) were administered highly pure HA (more than 97 %) at 200 mg/day or placebo for 12 months. During the trial, the patients were requested to conduct quadriceps-strengthening exercise daily as part of the treatment. The result reveals that quality of life according to the JKOM score was significantly improved in the HA group versus the placebo group (*p* < 0.05) and this trend was more obvious among patients aged ≤70 years (*n* = 21). For these relatively younger patients in the HA group, the total JKOM score was significantly better than that in the placebo group (*p* < 0.05). Thus, HA intake and quadriceps-strengthening exercise affectively alleviate knee pain particularly in patients aged ≤70 years (Fig. 3).

In 2013, Martinez-Puig et al. [42] reported that 50 healthy subjects with joint discomfort (VAS between 10 and 40 mm; HA group, *n* = 20; placebo group, *n* = 20, mean age, 59.6) were administered an HA mixture at 80 mg/day (HA content, 48 mg/day) or placebo for 90 days and outcomes were evaluated using an isokinetic dynamometer. Maximum peak torque was significantly greater in the HA group than in the placebo group (*p* < 0.05). In the HA group, the total knee extension and mean power of knee extension were also significantly higher than the values in the placebo group (*p* < 0.05). This trial demonstrated that oral HA effectively improves the ability of the knee joint to bend and stretch.

Moriña et al. [43] reported a meta-analysis of two randomized, controlled, double-blind, placebo-controlled trials involving a total of 148 patients with mild knee pain (VAS between 30 and 50 mm, aged from 20 to 75). In the studies, patients were administered an oral HA mixture at 80 mg/day (HA content, 48 mg/day) or placebo for 3 months and outcomes were evaluated using an isokinetic dynamometer. The analysis indicated that the affected joint displayed greater flexion in the HA group than in the placebo group (*p* = 0.039). The result of ultrasound examination for synovial effusion illustrated that synovial effusion was significantly reduced in the HA group (*p* = 0.029). An evaluation using a VAS indicated that knee pain was additionally improved in the HA group compared with the findings in the placebo group (*p* = 0.0036).

In 2014, Sánchez et al. [44] reported that 68 patients with mild knee pain (VAS between 30 and 50 mm; HA group, *n* = 34; placebo group, *n* = 34, mean age, 69.5) were administered an oral HA mixture at 80 mg/day (HA content, 48 mg/day) or placebo for 90 days. A significant improvement in knee pain according to the VAS was recorded in the HA group compared with the placebo group (*p* = 0.0005). An ultrasound examination indicated that synovial effusion was reduced in the HA group (*p* = 0.041). The result using an isokinetic dynamometer illustrated that muscular strength (peak torque) was significantly greater among patients in the HA group than among those in the placebo group (*p* = 0.0324). Blood tests for RNA expression were conducted in 20 patients (HA group, *n* = 10; placebo group, *n* = 10). The results demonstrated that the expression of genes related to glycosaminoglycan metabolism and extracellular matrix dynamics differed between the HA and placebo groups. It was concluded that differences in gene expression explained the improvements in knee pain and muscular strength in the HA group.

In 2015, Nelson et al. [45] treated 40 patients with knee OA (VAS > 50 mm; HA group, *n* = 21; placebo group, *n* = 19, mean age, 61.0) with an oral HA mixture at 80 mg/day (HA content, 56 mg/day) or placebo for 3 months. Significant improvements in the VAS, WOMAC total score, and WOMAC pain score were observed in the HA group compared with the placebo group (*p* < 0.05). An analysis of serum and synovial fluid illustrated that inflammatory cytokine levels were significantly increased in the placebo group (*p* < 0.05) and significantly decreased in the HA group (*p* < 0.05). Regarding bradykinin, which related to inflammation and pain, and leptin, which is related to OA caused by obesity, the levels of both substances were significantly lower in the HA group than in the placebo group (*p* < 0.05). Ten patients in each group drank large amounts of water to examine the turnover of HA in synovial fluid. The turnover rate was significantly decreased in the HA group compared with that in the placebo group (*p* = 0.046). Consequently, this report demonstrated that the oral intake of HA is useful for treating obese patients with OA.

In 2015, Jensen et al. [46] conducted a study in which 72 patients with knee OA (HA group, *n* = 37; placebo group, *n* = 35, mean age, 47.2) were administered a liquid HA mixture or placebo for 4 weeks. For the first 2 weeks, the HA mixture was given at 45 mL/day (HA content, 225 mg/day) and it was given at 30 mL/day (HA content, 150 mg/day) for the next 2 weeks. After 2 weeks of intake, knee pain as evaluated using a VAS was significantly improved in the HA group compared to baseline (*p* < 0.05). On the contrary, no improvement in knee pain was recorded in the placebo group. Thus, oral HA can relieve knee pain.

Among all the studies on clinical trials from 2008 to 2015, seven studies involved the treatment of the symptoms associated with synovitis, mainly pain [33–37, 39, 41, 46]; four involved measures taken to relieve synovial effusion or inflammation [38, 43–45]; and three involved measures taken to improve knee muscular strength [42–44].

The studies that used a mixture containing HA and other components could not rule out the possibility that the other components had effects on knee pain. However, studies that used highly pure HA (more than 97 %) also reported a beneficial effect on knee pain. Thus, oral HA can effectively improve knee pain.

It is apparent that there were slightly more female subjects than males investigated in the above-mentioned clinical studies (mean ratio: males 43 %, females 57 %). However, each study involved the same gender ratio for HA and placebo groups. In general, the number of female OA patients was larger than the number of males. This is probably because females have lesser muscles than males and therefore, the burden on the knee increases. It has been reported that HA is regulated by ovarian steroids [47]; however, it is not yet clear whether the oral intake of HA influences hormonal balance. This needs further research.

HA intake differs among the respective studies, such as 48–240 mg/day for 2 weeks–12 months. Thus, further research is needed to clarify the minimal effective dose and the minimum intake period of HA.

## Safety of HA

Chicken comb, which contains highly concentrated HA has long been consumed in Germany, France, and China. In Japan, HA dietary supplements have been available since 1992. HA is approved as a healthy raw material of new resource food in China, as a food additive and healthy functional food in Korea, and as a food additive in Japan. In addition, HA is sold globally in countries such as the US, Canada, Italy, and Belgium and no adverse event caused by HA has been reported.

Several safety studies of HA have been conducted (Table 2). No toxicity was observed in single-dose toxicity studies [48–50], repeated-dose toxicity studies [51–61], reproductive and developmental toxicity studies [62–69], mutagenicity tests [70–74], and antigenicity studies [75, 76]. It is reported that exogenous HA such us that administered orally is not harmful to human cancer cells [77]. A 12 month clinical study identified no adverse event attributable to HA [41]. Orally administrated HA is used by body tissues and approximately 90 % of exogenous HA is metabolized and released in expiration and urine; thus, the polysaccharide does not accumulate at excessive levels in the body [78]. There were no adverse events attributable to HA in any of the aforementioned clinical trials [34–44]. Based on these factors, HA can be considered as a safe food material.

**Table 2:** Safety tests of hyaluronan

Test procedures		Subjects	Route	Results	References
Randmized, double-blind, placebo-controlled trial		Human	Oral administration, 60 mg/day for 2 weeks	No adverse event related to hyaluronan.	[34]
		Human	Oral administration, 48 mg/day for 2 months	No adverse event related to hyaluronan.	[35]
		Human	Oral administration, 240 mg/day for 8 weeks	No adverse event related to hyaluronan.	[36]
		Human	Oral administration, 200 mg/day for 8 weeks	No adverse event related to hyaluronan.	[37]
		Human	Oral administration, 60 mg/day for 4 months	No adverse event related to hyaluronan.	[39]
		Human	Oral administration, 200 mg/day for 12 months	No adverse event related to hyaluronan.	[41]
		Human	Oral administration, 52 mg/day for 3 months	No adverse event related to hyaluronan.	[42]
		Human	Oral administration, 52 mg/day for 3 months	No adverse event related to hyaluronan.	[43]
		Human	Oral administration for 4 weeks (225 mg/day for first 2 weeks, 150 mg/day for last 2 weeks)	No adverse event related to hyaluronan.	[46]
single-dose toxicity study		Mouse	Oral administration	LD50 (mg/kg) > 2400	[48]
		Mouse	Oral administration	LD50 (mg/kg) > 500	[49]
		Rat	Oral administration	LD50 (mg/kg) > 800	[48]
		Rat	Oral administration	LD50 (mg/kg) > 200	[50]
		Rabbit	Oral administration	LD50 (mg/kg) > 1000	[48]
Repeated-dose toxicity study		Rat	Subcutaneous administration for 13 weeks with 4 weeks recovery test	NOAEL 50 mg/kg/day	[51]
		Beagle dog	Subcutaneous administration for 13 weeks with 4 weeks recovery test	NOAEL 10 mg/kg/day	[52]
		Rat	Oral administration for 30 days	NOAEL 1500 mg/kg/day	[53]
		Rat	Oral administration for 90 days	NOAEL 1333 mg/kg/day	[54]
		Rat	Oral administration for 90 days	NOAEL 1000 mg/kg/day	[55]
		Rat	Oral administration 28 days	NOAEL 3500 mg/kg/day	[56]
		Rat	Intraperitoneal administration 90 days	NOAEL 9 mg/kg/day	[57]
		Rat	Intraperitoneal administration for 3 months	NOAEL 60 mg/kg/day	[58]
		Rat	Oral administration for 13 weeks	NOAEL 12.5 mg/kg/day	[59]
		Rat	Oral administration for 90 days with 28 days recovery test	NOAEL 48 mg/kg/day	[60]
		Beagle dog	Intra-articular administration for 6 months	NOAEL 12 mg/kg/day	[61]
Reproductive and developmental toxicity studies		Rat	Subcutaneous administration	NOAEL 50 mg/kg/day	[62–64]
		Rat	Oral administration	NOAEL 670 mg/kg/day	[65]
		Rat	Subcutaneous administration	NOAEL 50 mg/kg/day	[66–68]
		Rabbit	Subcutaneous administration	NOAEL 50 mg/kg/day	[69]
Mutagenicity test	Reverse mutation test	Bacteria(Ames test)	1000 μg/plate	Negative	[70]
	Chromosomal aberration test	Mammalian cultured cell	1.00 mg/mL	Negative	[71]
		Mammalian cultured cell	1000 μg/plate	Negative	[72]
	Micronucleus test	Mouse	300 mg/kg	Negative	[73]
		Mouse	Intraperitoneal administration, 30 mg/kg	Negative	[74]
Antigenicity study		Mouse, Rat	Intraperitoneal administration, 100 μg/個体	Negative	[75]
		Guinea pig		Negative	
		Rabbit	Intramuscularl administration, 30 mg/kg	Negative	[76]
Influence on cancer cell		Mouse, cell	Oral administration, 200 mg/day	No influence on cancer cell	[77]

## Conclusion

The population is aging rapidly around the world. In addition to medical treatment, self-medication is more commonly practiced to reduce patient burden and enhance QOL. HA is a safe raw material and the efficacy of oral HA in relieving knee pain was demonstrated in several clinical trials. HA as a dietary supplement exhibits mild efficacy and no side effects. By utilizing these characteristics, HA dietary supplements provide at least some possibility for the treatment and prevention of serious conditions in patients with OA exhibiting mild knee pain. This review may improve the understanding of HA dietary supplements and it is expected that HA will emerge as a modality for treating knee pain that can be safely used by patients.

## Abbreviations

HA: hyaluronan
IL-10: interleukin-10
JKOM: the Japanese Knee Osteoarthritis Measure
LD50: lethal dose 50 %
MW: mean molecular weight
N/A: Not available
NOAEL: No Observed Adverse Effect Level
OA: osteoarthritis
PCT: paracetamol
SF-36v2: the Short Form-36 Acute US Version 2
SOCS3: Suppresor Of Cytokine Signaling3
TLR-4: Toll-Like Recepter 4
WOMAC: The Western Ontario and McMaster Universities Osteoarthritis Index
